# Functional Evolution of Duplicated Odorant-Binding Protein Genes, *Obp57d* and *Obp57e*, in *Drosophila*

**DOI:** 10.1371/journal.pone.0029710

**Published:** 2012-01-06

**Authors:** Eriko Harada, Jun Nakagawa, Tsunaki Asano, Masato Taoka, Hiroyuki Sorimachi, Yoshihiro Ito, Toshiro Aigaki, Takashi Matsuo

**Affiliations:** 1 Department of Biological Sciences, Tokyo Metropolitan University, Hachioji, Tokyo, Japan; 2 Department of Molecular Developmental Biology, Tokyo Metropolitan Institute of Medical Science, Kamikitazawa, Tokyo, Japan; 3 Department of Chemistry, Tokyo Metropolitan University, Hachioji, Tokyo, Japan; 4 Calpain Project, Tokyo Metropolitan Institute of Medical Science, Kamikitazawa, Tokyo, Japan; 5 Nano Medical Engineering Laboratory, RIKEN Advanced Science Institute, Hirosawa, Saitama, Japan; 6 Department of Agricultural and Environmental Biology, University of Tokyo, Yayoi, Tokyo, Japan; AgroParisTech, France

## Abstract

Odorant-binding proteins (OBPs) are extracellular proteins found in insect chemosensilla, where they participate in the sensing of odors, tastes, and pheromones. Although a large number of OBP genes have been identified in insect genomes, their molecular functions and biological roles have been clarified in limited cases. Two OBP genes, *Obp57d* and *Obp57e*, were involved in the evolution of host-plant preference in *Drosophila sechellia*. Comparative analyses of the *Obp57d/e* genomic sequences from 27 closely related species suggested that the two genes arose by tandem gene duplication and functionally diverged from each other. In this study, the functional evolution of *Obp57d* and *Obp57e* was examined by *in vitro* binding assays using recombinant proteins synthesized in a bacterial system. Compared to the ancestral Dpse\OBP57de, Dmel\OBP57d was more specialized to tridecanoic acid while Dmel\OBP57e was generalized regarding their binding affinity, suggesting that the two OBP genes underwent subfunctionalization and neofunctionalization. A behavioral analysis using knockout flies supported that the biological role is different between OBP57d and OBP57e *in vivo*. Site-directed mutagenesis of the evolutionarily conserved amino acids revealed that these residues play an important role in protein folding. These findings provide a clue to understanding how the repertoire of OBP genes is maintained in a genome under natural selection.

## Introduction

In insects, olfaction and gustation play an important role in the detection of foods, egg-laying substrates, mates and predators. The sensing of chemical compounds is enabled by chemosensory receptors, including odorant receptors (ORs) and gustatory receptors (GRs). The genes for these receptors comprise a large multigene family in a genome, corresponding to a wide variety of chemical compounds to be sensed. Besides the receptors, odorant-binding proteins (OBPs) also function in the peripheral chemosensory system of insects by interacting with chemical compounds at the initial step of perception. In *Drosophila*, about 50 OBP genes have been identified to form a gene family in a genome [1], [2], comparable in the size to that of receptor genes (about 60 each for ORs and GRs) [3]–[6], indicating that they too contribute to the discrimination of chemical compounds.

OBPs are small, soluble proteins expressed at a high concentration in the lymph filling chemosensilla, where they are thought to bind to and solubilize hydrophobic compounds. Several OBPs have been shown *in vitro* to interact with ecologically important compounds. For example, *Acyrthosiphon pisum* OBP3 was shown to interact with its alarm pheromone, (E)-β-Farnesol [7]. Two OBPs from *Anopheres gambiae*, OBP1 and OBP4, were shown to bind to indole, a component of human body odor [8]–[10]. In *Culex pipiens*, OBP1 was identified to interact with the oviposition pheromone, 6-acetoxy-5-hexadecanolide [11]. Independent models were proposed for the biological role of each OBP in chemosensation. A pheromone-binding protein (PBP) of the gypsy moth *Lymantria dispar*, PBP2, is thought to function as a scavenger [12], [13]. Another PBP in the silkworm moth *Bombyx mori*, PBP1, is considered as a transporter for the ligand, bombykol [14]. In the case of a *Drosophila* OBP, LUSH (OBP76a), the integrated analyses of behavioral genetics, protein crystallography, and electrophysiology showed that the conformational change of LUSH protein on binding to *cis*-vaccenyl acetate is necessary for activation of the corresponding receptor, OR67d [15]. These studies successfully demonstrated that an integrated approach using both *in vitro* and *in vivo* analyses is required for a thorough understanding of the biological roles of OBPs.

Two OBP genes, *Obp57d* and *Obp57e*, were identified to be involved in the evolution of host-plant preference in *Drosophila sechellia* [16]. In *D. melanogaster*, *Dmel\Obp57d* and *Dmel\Obp57e* were co-expressed in the taste sensilla on the legs, contributing to the taste sensation of octanoic acid, a toxin contained in the host plant of *D. sechellia* [17], [18]. Comparative analyses of the *Obp57d/e* locus among 27 *Drosophila* species showed that *Obp57d* and *Obp57e* arose by gene duplication of an ancestral gene, which remains as a single gene, *Dpse\Obp57de*, in *D. pseudoobscura* [19]. Although the amino acid sequences of OBP57d and OBP57e are highly diverged, 16 sites were conserved among species, as well as between OBP57d and OBP57e, suggesting that these residues are functionally important.

In this study, the functional evolution of *Obp57d* and *Obp57e* was examined by an integrated approach using the *in vitro* binding assay and the *in vivo* behavioral analysis. Moreover, the role of the evolutionary conserved residues was examined using site-directed mutagenesis. The results showed that the two OBPs differ in the ligands recognition and their biological roles, suggesting that functional differentiation after gene duplication was the evolutionary driving force for *Obp57d* and *Obp57e*.

## Results

### Expression and purification of recombinant OBPs in *E. coli*

**Figure 1:**
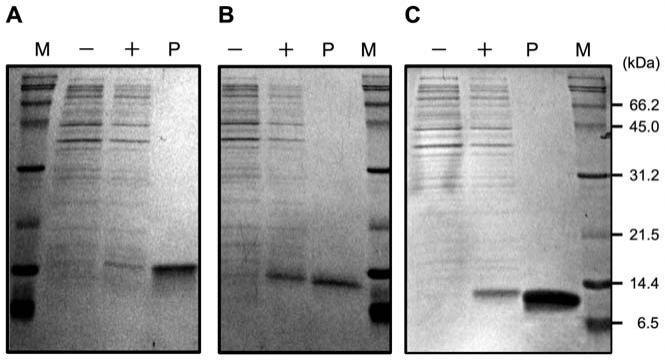
Expression and purification of recombinant OBPs. Bacterial cells before (−) and after (+) induction by IPTG, and purified protein (P) were analyzed by SDS-PAGE. (A) Dmel\OBP57d, (B) Dmel\OBP57e, and (C) Dpse\OBP57de. The expected size of the expressed OBPs is about 13 kDa.

Several methods have been used for the expression of recombinant OBPs in the *E. coli* system. We first tried the periplasmic expression using the vector pET26b [11], [14], [20]–[22]. Although various conditions were explored, neither Dmel\OBP57d, Dmel\OBP57e, nor Dpse\OBP57de was expressed in the periplasmic fraction, suggesting that these OBPs are not compatible with this method (data not shown). Therefore, the pET30b expression vector and the BL21(DE3) host cell was used and the expressed recombinant OBPs were recovered from the insoluble cytoplasmic fraction (inclusion bodies). All three OBPs were expressed with high efficiency (4.9, 12.5, and 10.5 mg/L of culture for Dmel\OBP57d, Dmel\OBP57e, and Dpse\OBP57de, respectively) (Fig. 1).

Many OBPs have been successfully denatured and refolded using the established method [7], [9], [23]. However, this was not the case for our OBPs, resulting in the formation of nonspecific multimers probably due to the formation of inappropriate disulfide bonds. Several factors were critical for the correct refolding of OBP57d and OBP57e. First, denaturing agents affected the refolding step. Guanidine hydrochloride (GdnHCl) provided good results for Dmel\OBP57d and Dpse\OBP57de, but not for Dmel\OBP57e, for which urea was used. Second, the denaturing agents were removed by dilution against the refolding buffer, which gave the best result at pH 9.4 instead of pH 7.4. The addition of GSH/GSSG at a ratio of 1∶10 promoted the formation of the monomer during the refolding step (data not shown).

The refolded proteins were purified by anion exchange chromatography followed by gel filtration, according to an established method [23]. Monomeric OBP was eluted in the middle fractions of the 0–0.5 M NaCl gradient and separated from multimeric OBP eluted in the later fractions (Fig. S1A–C). The fractions containing monomeric OBP were subjected to gel filtration and used in subsequent experiments.

The purity of the recombinant OBPs was examined by native-PAGE and HPLC (Fig. S1D, S2). Correct refolding of the purified OBPs was confirmed by various methods. The formation of α helices was confirmed by using CD spectrometry (Fig. S3A). The formation of disulfide bonds between specific cysteine residues was examined by peptidase digestion followed by mass spectrometry (Table S1). The predicted disulfide bonds were confirmed in Dmel\OBP57e and Dpse\OBP57de, while two alternative possibilities remained in Dmel\OBP57d. Nevertheless, regarding the results of native-PAGE and HPLC, a single type of monomeric Dmel\OBP57d was obtained by the same method used for Dpse\OBP57de, suggesting that the purified Dmel\OBP57d consists of homogenous molecules which presumably represents the correct folding.

### *In vitro* binding assay using intrinsic fluorescence

**Figure 2:**
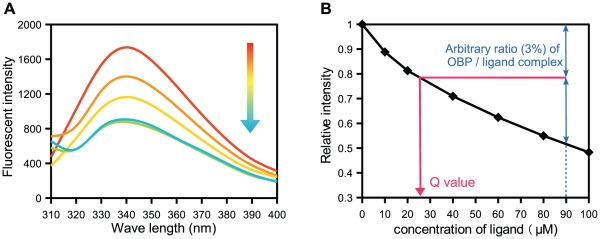
*In vitro* binding assay using intrinsic fluorescence. The intrinsic fluorescence spectrum from tryptophan residues was recorded between 300 and 400 nm. (A) The fluorescence intensity was decreased by increasing the concentration of ligands. The data on the interaction between Dmel\OBP57d and tridecanoic acid is shown. An arrow indicates the direction of change observed as the ligand concentrations increased. (B) The relative fluorescence intensity at 340 nm was plotted against concentrations of ligands (solid black line). For each OBP, the relationship between relative intensity and the actual amount of bound ligand was determined by the quantitative binding assay using 90 µM methyl tridecanoate (shown in blue). The quenching value (Q) was defined as the concentration of ligand at which 3% of 1 µM OBP molecules are bound by the ligand, assuming a 1∶1 association between the OBP and ligand (shown in red).

The competitive binding of fluorescent dyes is widely used to study the interaction between OBPs and small organic compounds *in vitro* [7], [9], [11], [13], [21], [23], [24]. However, this method could not be applied to Dmel\OBP57d, Dmel\OBP57e, and Dpse\OBP57de because the fluorescent probes, such as N-phenyl-1-naphthylamine (1-NPN) and 1-aminoanthracene (1-AMA), did not bind to these OBPs (data not shown). Therefore, the intrinsic fluorescence from tryptophan was used to monitor the interaction [13]. The intensity of the fluorescence from tryptophan varies depending on the surrounding environment. It was reported for a Bovine binding protein that the fluorescence intensity of the tryptophan residue located inside of the binding pocket was altered on binding to its ligand [25]. In fact, in all three OBPs we analyzed, addition of the putative ligand quenched the intrinsic fluorescence in a concentration-dependent manner (Fig. 2A), showing that this method can be used to monitor the interaction between these OBPs and ligands.

Compared with that of other OBPs, the binding of Dmel\OBP57d, Dmel\OBP57e, and Dpse\OBP57de differs in two ways. First, when a concentration-dependent change in fluorescence was observed in the scatchard plot, the data points did not align linearly, suggesting that the binding kinetics is different between lower and higher concentrations of a ligand. Because a dissociation constant could not be calculated, a quenching value (Q) was used to compare the binding affinity among various compounds (Fig. 2B; see below). Second, the response to acidity is different from that of other OBPs. Previous studies found that, in many cases, OBPs show higher affinity to ligands at around a neutral pH (for example pH 7.0 in BmorPBP1 and CpipOBP1) than around a lower pH [11], [14], [20], [22], [26]–[28]. Contrary to those observations, in Dmel\OBP57d, Dmel\OBP57e, and Dpse\OBP57de, the affinity to a ligand was higher at pH 5.0 than pH 7.4 (Fig. S3B). The CD spectral analysis showed that there was no difference in secondary structure between pH 7.4 and 5.0 (Fig. S3A).

**Figure 3:**
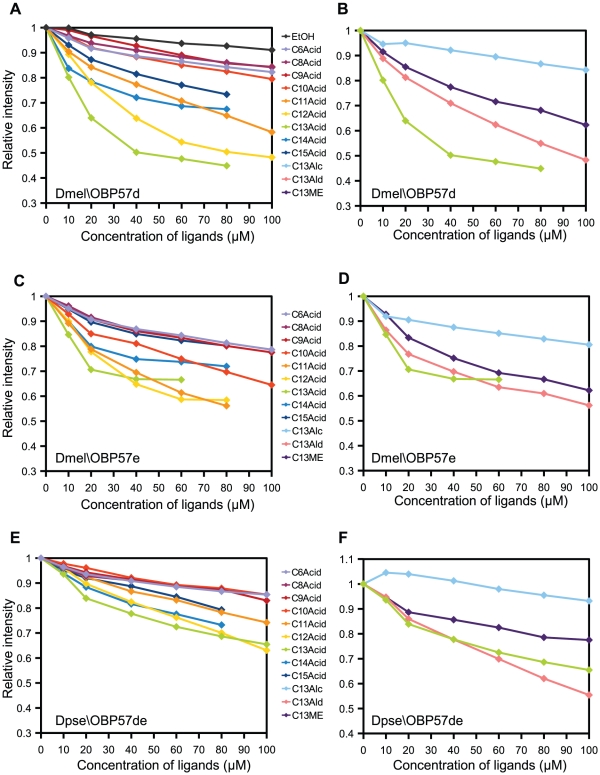
Binding affinity of the recombinant OBPs to various compounds. The binding affinity to C6, C8–15 fatty acids and C13 compounds with different functional groups was examined by the *in vitro* binding assay using intrinsic fluorescence. (A, B) Dmel\OBP57d, (C, D) Dmel\OBP57e, and (E, F) Dpse\OBP57de. The ligand solution was added to a 1 µM OBP solution. The Y axis indicates the relative fluorescence intensity at 340 nm. Mean values of three independent replicates are shown. C13Alc, C13Ald, and C13ME indicate 1-tridecanol, 1-tridecanal, and methyl tridecanoate, respectively.

Dmel\OBP57d and Dmel\OBP57e were identified to be involved in the taste perception of octanoic acid [16], [18]. To investigate their binding specificity, a series of fatty acids were screened for their affinity to Dmel\OBP57d by using the *in vitro* binding assay. To our surprise, the strongest interaction was observed with the longer chain fatty acid (tridecanoic acid: C13), with gradually decreasing affinity to longer or shorter chain fatty acids (Fig. 3A). The binding affinity to other C13compounds with different functional groups was also examined. The binding affinity to 1-tridecanol, 1-tridecanal and methyl tridecanoate was weaker than that to tridecanoic acid (Fig. 3B), suggesting that Dmel\OBP57d has the highest affinity to acids. Binding affinity to the known ligands for other insect OBPs was also tested. Hexyl benzoate (HB) is a ligand for ApisOBP3 [7], 2-pentadecanone (2PO) is a ligand for *Locusta migratoria* OBP1 [24], and linalool (LL) is an attractant for *Drosophila* larvae [29]. The binding affinity to these compounds was lower than that to tridecanoic acid (Fig. S4), again supporting that Dmel\OBP57d specifically recognizes fatty acids.

**Figure 4:**
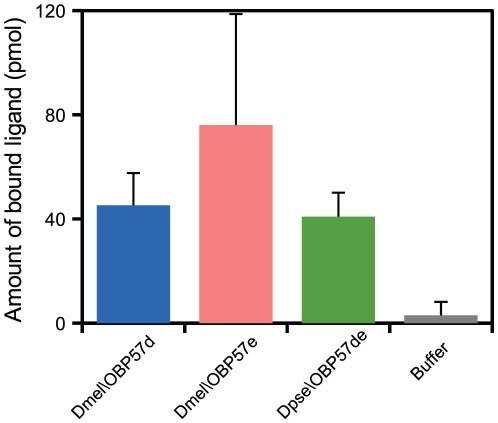
Direct quantification of bound ligand. Methyl tridecanoate was added to 1 mL of 1 µM OBP solution at a concentration of 90 µM. After ultrafiltration and extraction, the amount of the bound ligand was directly determined by GC-MS. Bars represent the means of three independent replicates, and error bars indicate standard error.

**Figure 5:**
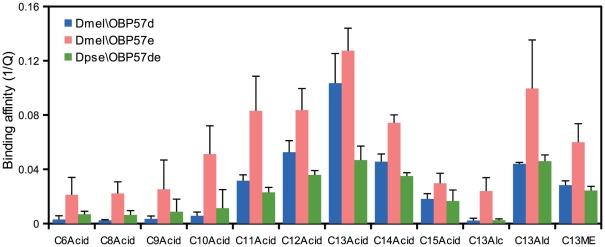
Comparisons of the binding specificity among Dmel\OBP57d, Dmel\OBP57e, and Dpse\OBP57de. Binding affinity to various ligands was compared among the three OBPs using the quenching value (Q, see Figure 2). A higher 1/Q value means higher affinity to the ligand. Bars represent the means of three independent replicates, and error bars indicate standard error.

The binding affinity of Dmel\OBP57e and Dpse\OBP57de to the same set of compounds was examined (Fig. 3C–F). The interaction-dependent decrease in the intrinsic fluorescence was normalized to the amount of bound ligand directly quantified by using GC-MS (Fig. 2B, 4; see Materials and Methods S1) [12], [26], to compare the binding affinity to the same ligand among the three OBPs. The overall binding affinity of Dpse\OBP57de was similar to that of Dmel\OBP57d, except for tridecanoic acid to which Dmel\OBP57d showed much higher affinity (Fig. 5), indicating that Dmel\OBP57d acquired higher specificity to tridecanoic acid after gene duplication. Dmel\OBP57e also showed higher affinity than Dpse\OBP57de, not only to tridecanoic acid but also to other compounds (Fig. 5). This increase of affinity was not proportional among compounds; the increase was particularly obvious in the affinity to C10–C13 fatty acids and 1-tridecanol. For example, the increase was 10-fold for 1-tridecanol while it was twofold for methyl tridecanoate.

### Oviposition site selection assay

**Figure 6:**
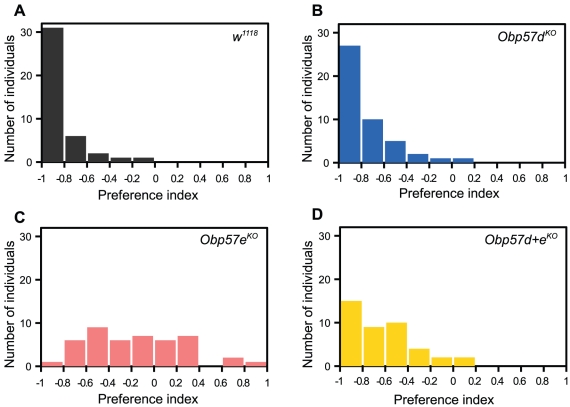
Behavioral response to tridecanoic acid in the OBP mutants. Behavioral responses of the *D. melanogaster* knockout flies for *Obp57d* and *Obp57e* to tridecanoic acid were examined by the oviposition site selection assay. Preference index (PI) = (*N_acid_*−*N_cont_*)/(*N_acid_*+*N_cont_*), where *N_acid_* and *N_cont_* are the number of eggs laid on tridecanoic acid-containing and control media, respectively. A total of 48 individuals were examined in the six independent replicates. (A) *w^1118^*, (B) *Obp57d^KO^*, (C) *Obp57e^KO^*, and (D) *Obp57d+e^KO^*.

Dmel\OBP57d and Dmel\OBP57e were shown to have the highest affinity to tridecanoic acid by the *in vitro* binding assay. To examine the biological significance of this finding *in vivo*, the behavioral response of the *D. melanogaster* mutants for *Obp57d* and *Obp57e* (*Obp57d^KO^*, *Obp57e^KO^*, and *Obp57d+e^KO^*) to tridecanoic acid was examined by the oviposition site selection assay. *D. melanogaster* wild-type flies completely avoided the media containing tridecanoic acid (Fig. 6), suggesting that tridecanoic acid acts as a repellent as octanoic acid does [18]. The difference among strains was statistically significant by the Kruskal-Wallis test (*χ^2^* = 76.146, *n* = 3, *P*<2.2e^−16^), and the difference between each pair of strains was further analyzed by the pairwise Wilcoxon rank sum test (Table 1). Although there was no significant difference between *Obp57d^KO^* and wild-type flies, *Obp57e^KO^* flies showed significantly reduced avoidance, suggesting that Dmel\OBP57e is required for the efficient sensing of tridecanoic acid. Interestingly, *Obp57d+e^KO^* flies showed an intermediate phenotype between that of the wild-type and *Obp57e^KO^* flies, suggesting that Dmel\OBP57d acts inhibitory to the tridecanoic acid sensing in the absence of Dmel\OBP57e. Taken together, both OBP57d and OBP57e are involved in the behavioral response to tridecanoic acid presumably by interacting with each other.

**Table 1:** p-values of the pairwise Wilcoxon rank sum test with adjustment by Holm's method for multiple comparisons for the results of the oviposition site selection assay.

	*w^1118^*	*Obp57d^KO^*	*Obp57e^KO^*
***Obp57d^KO^***	0.0737		
***Obp57e^KO^***	8.9e^−12^	1.5e^−10^	
***Obp57d+e^KO^***	1.4e^−5^	0.0030	2.5e^−06^

### Functional importance of the amino acids conserved between *Obp57d* and *Obp57e*

**Figure 7:**
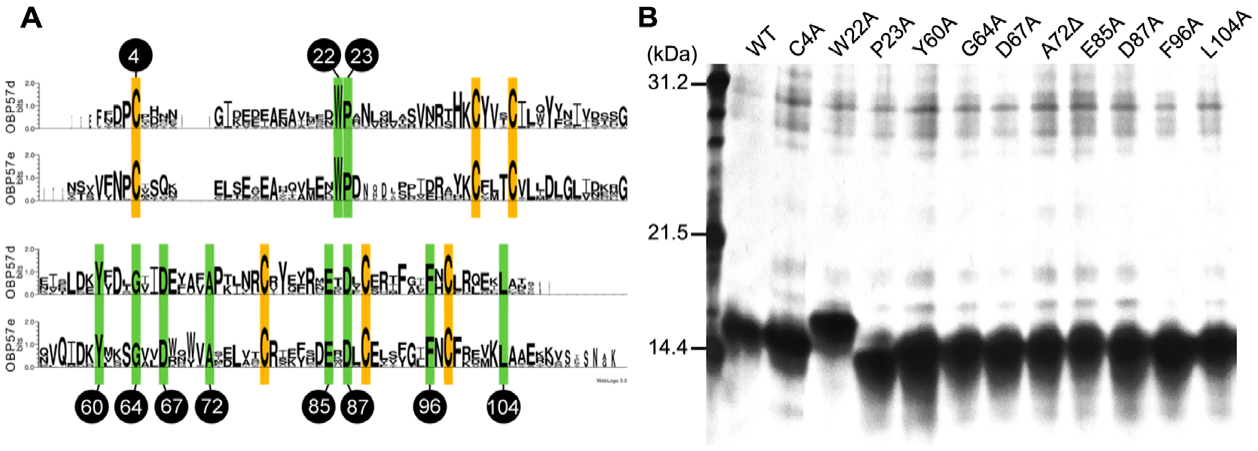
Site-directed mutagenesis of the evolutionarily conserved amino acids. (A) Aligned sequence Logo representation of OBP57d and OBP57e. Among the 16 evolutionarily conserved sites (highlighted by yellow for the OBP signature cysteins and green for the others) [19], 11 were selected for site-directed mutagenesis in Dmel\OBP57d (indicated by black circles; numbers indicate the position in Dmel\OBP57d). (B) Expression of the 11 mutated OBPs in the insoluble cytoplasmic fraction was confirmed by SDS-PAGE.

Evolutionary comparisons of OBP57d and OBP57e sequences revealed that amino-acid residues at 16 sites were highly conserved among 27 *Drosophila* species [19] (Fig. 7A). These sites are expected to be important for OBP functions. To examine this possibility, a series of mutated forms of Dmel\OBP57d was generated for eleven of the 16 sites by site-directed mutagenesis, and their binding affinity was analyzed by the *in vitro* binding assay.

**Figure 8:**
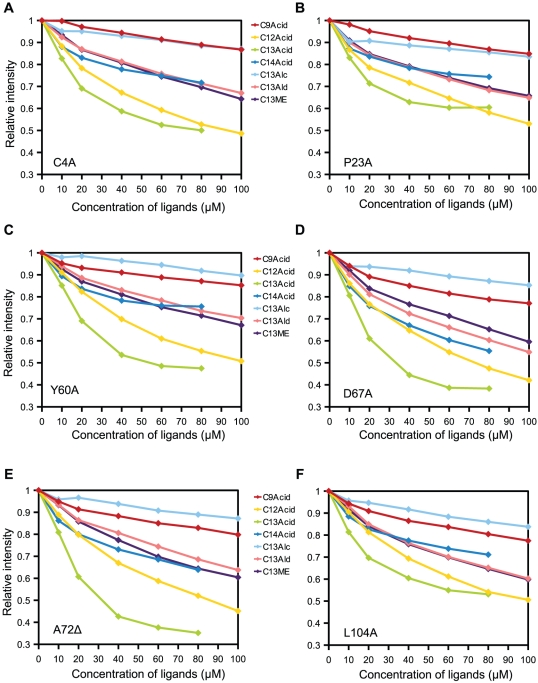
Binding affinity of the mutated Dmel\OBP57d. The binding affinity of six mutated forms of Dmel\OBP57d was examined by the *in vitro* binding assay using intrinsic fluorescence. (A) C4A, (B) P23A, (C) Y60A, (D) D67A, (E) A72Δ, and (F) L104A. C13Alc, C13Ald, and C13ME indicate 1-tridecanol, 1-tridecanal, and methyl tridecanoate, respectively.

**Figure 9:**
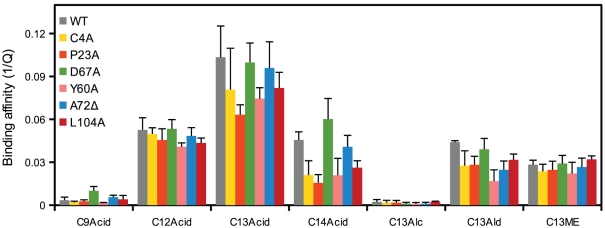
Comparisons of binding specificity among the mutated forms. Binding affinity to various ligands was compared using the quenching value (Q), as shown in Figure 5. Bars represent the means of three independent replicates, and error bars indicate standard error.

The mutated OBPs were expressed and purified by the same method used for the wild-type Dmel\OBP57d. All of them were expressed efficiently the in insoluble cytoplasmic fraction (Fig. 7B). However, five of the 11 forms were not refolded successfully, forming soluble but nonspecific multimers, suggesting that these sites were important for correct folding of the protein (data not shown). For the remaining six forms, soluble monomers were purified. The binding affinity of these proteins to fatty acids and other C13 compounds with different functional groups was examined (Fig. 8). Compared to the wild-type OBP57d, each of the six mutated OBPs showed a reduction or increase in affinity to particular compounds. However, none showed a complete loss of interaction with ligands (Fig. 9), suggesting that once refolded in an appropriate structure, these OBPs are capable of expressing some binding activity, though their affinity is reduced.

## Discussion

This is the first report describing the expression of recombinant Dmel\OBP57d, Dmel\OP57e, and Dpse\OBP57de. Consistent with other OBPs, three disulfide bonds were confirmed to be conserved in all three OBPs. Although the optimal conditions for refolding were different, the conditions used in the purification were similar to those used for other OBPs, suggesting that the basic structure and biochemical character of the refolded proteins were conserved.

Many OBPs are known to change their conformation depending on the acidity of the surrounding environment, which is thought to be important for their association-dissociation kinetics [14], [22], [28]. The intrinsic fluorescence of Dmel\OBP57d, Dmel\OBP57e and Dpse\OBP57de decreased with increasing acidity (data not shown), but the CD spectral analysis showed that their secondary structure was still maintained (Fig. S3A), suggesting that these OBPs change their conformation depending on the pH while maintaining their secondary structure as reported for other OBPs. The possibility that acidity affected the interaction by changing the solubility of compounds can be excluded, because pH-dependent changes in the strength of the interaction were observed even with methyl esters. Therefore, higher affinity to the ligands at pH 5.0 seemed to be an unique feature of Dmel\OBP57d, Dmel\OBP57e and Dpse\OBP57de.

The *in vitro* binding assay using intrinsic fluorescence showed that Dmel\OBP57d and Dmel\OBP57e strongly interact with fatty acids, with the highest affinity to tridecanoic acid. These fatty acids are smaller than the known ligands for other OBPs, suggesting that the binding pocket of Dmel\OBP57d and Dmel\OBP57e is smaller than that of other OBPs. This might explain why these OBPs did not bind to 1-NPN, a fluorescent probe widely used for the *in vitro* binding assay.

It was also revealed that Dmel\OBP57d, Dmel\OBP57e and Dpse\OBP57de interacted with ligands at relatively high concentrations (>10 µM) compared to other OBPs, which interacted at concentrations below 5 µM. The differences in kinetics might reflect the fact that OBP57d and OBP57e are expressed in the taste sensilla and participate in the gustatory sensation [18], because taste neurons are normally activated by ligands at concentrations as high as mM. In fact, the behavioral response to octanoic acid and tridecanoic acid was observed at concentrations of between 0.5 and 4 mM [Matsuo, unpublished data].

The ecological significance of avoiding tridecanoic acid in *Drosophila* is not known. Fatty acids and alkanes are used as energy sources by bacteria, fungi and yeast under the conditions where nutrients are limited. Tridecanoic acid is directly utilized as a metabolite by microorganisms, or produced as an intermediate metabolite from pentadecanoic acid or hexadecane [30]–[33]. Such metabolic pathways are utilized by entomopathogenic microorganisms including *Bauveria bassiana*, degrading hydrocarbons and fatty acids on the epidermis [34]. Because tridecanoic acid is rare in the environment, it existence would be a sign of contamination by harmful microorganisms. If so, the sensing of tridecanoic acid would be important for *Drosophila* to prevent the larvae from being infected, assuring the flora of their reproductive sites, ripe and fermented fruits.

In the previous study, comparative analyses showed that particular amino acids were highly conserved between OBP57d and OBP57e, in contrast to the other sites showing extremely high evolution rate [19]. Site-directed mutagenesis revealed that half of the conserved sites were important for appropriate protein folding, suggesting that these residues were conserved under selective pressure to maintain the fundamental structure shared between OBP57d and OBP57e.

We have experimentally reconstructed the functional evolution of *Obp57d* and *Obp57e* in ligand recognition. Compared to the ancestral Dpse\OBP57de, Dmel\OBP57d has higher affinity to tridecanoic acid, while Dmel\OBP57e has increased affinity not only to tridecanoic acid but also to other compounds. These results suggest that a combination of subfunctionalization and neofunctionalization after gene duplication was the evolutionary driving force for these OBPs; Dmel\OBP57d was more specialized to tridecanoic acid, while Dmel\OBP57e was generalized. Such subfunctionalization/neofunctionalization after gene duplication might also contribute to the evolution of other OBP genes.

In addition to the binding properties, the behavioral analysis suggested that the biological roles of Dmel\OBP57d and Dmel\OBP57e are different from each other. Dmel\OBP57e was involved in the avoidance of tridecanoic acid (Fig. 6C), probably by contributing to the enhanced sensitivity to the compound as a transporter. On the other hand, because the behavioral response of *Obp57d^KO^* flies was similar to that of wild-type flies (Fig. 6A, B), the contribution of Dmel\OBP57d to the avoidance of tridecanoic acid might be small. Along with these results, two lines of evidence suggest that other factors, as well as *in vitro* binding affinities, should be considered for thorough understanding of the biological roles of these OBPs. First, the expression levels of *Dmel\Obp57d* and *Dmel\Obp57e* are different from and dependent to each other [16]. The expression level of *Dmel\Obp57e* was 5 times higher than that of *Dmel\Obp57d* in wild-type flies, suggesting that the contribution of Dmel\OBP57d is smaller than that of Dmel\OBP57e. Moreover, the expression of *Dmel\Obp57e* was increased in *Obp57d^KO^* flies by approximately 10-fold, raising the possibility that the overexpression of Dmel\OBP57e might have compensated for the loss of Dmel\OBP57d in *Obp57d^KO^* flies. Second, downstream receptors for Dmel\OBP57d and Dmel\OBP57e, might be different from each other. It has been shown that *Dmel\Obp57d* is involved in courtship behavior while *Dmel\Obp57e* is not [35]. A gustatory receptor, GR32a, has been proposed as the receptor responsible for this behavior, being a possible downstream component of Dmel\OBP57d. The receptor responsible for oviposition behavior has not been identified. Recently, one of the ionotropic receptors (IRs), IR64a, was shown to be involved in the acid sensing by the *Drosophila* olfactory system [36]. If some IRs are expressed in the gustatory system, they might be strong candidates for the downstream component of Dmel\OBP57e as the fatty acid receptors. Since OBPs are secreted proteins, they can access multiple neurons housed in the same sensilla. The difference in the biological roles between Dmel\OBP57d and Dmel\OBP57e suggests that subfunctionalization of OBPs occurs also in the selectivity for the downstream receptors with which they functionally interact.

Although the downstream receptors might differ between Dmel\OBP57d and Dmel\OBP57e, the behavioral response of the *Obp57e^KO^* and *Obp57d+e^KO^* flies indicates that Dmel\OBP57d has an inhibitory effect on the sensing of tridecanoic acid. This effect could be, for example, explained by the titration of the ligand by Dmel\OBP57d. Nevertheless, little is known about the interaction between Dmel\OBP57d and Dmel\OBP57e; Is it direct or indirect? Do they function cooperatively or competitively? These questions must be answered by further analysis using an integrated approach involving biochemistry, neurophysiology, and behavioral genetics.

## Materials and Methods

### Chemicals

Hexanoic acid, decanoic acid and hexyl benzoate were purchased from Wako Pure Chemical Industries (Japan), and 2-pentadecanone, from Sigma (USA). Methyl tridecanoate, 1-tridecanol, 1-tridecanal, and myristic acid were purchased from Tokyo Chemical Industry (Japan). The other chemicals were obtained from Kanto Chemical (Japan). All the chemicals were of the highest grade available.

### cDNA cloning and construction of the vector plasmids

To obtain the OBP cDNAs, total RNA was extracted from the legs of 20 staged females with the QIAshredder and RNeasy Micro kit (QIAGEN, USA), and cDNA was synthesized using the SuperScript III first strand synthesis system (Invitrogen, USA) with the oligo(dT)20 primer. PCR was carried out by using ExTaq (Takara, Japan) with the following primer pairs: 5′-CCAACGATCCGTGCCCCCATA-3′ and 5′-AAACTCGAGTTATGACTTTGTTAATATTTCTTGCC-3′ for *Dmel\Obp57d*, 5′-CCAACACTTCAGTATTTAATCCGT-3′ and 5′-AAACTCGAGCTACTTTGCATTACTAATTGAAAC-3′ for *Dmel\Obp57e*, and 5′-CCCACAGTAATACTGCAATA-3′ and 5′-AAACTCGAGTCATTCCCAAGTGGTCGCTG-3′ for *Dpse\Obp57de*. The amplified fragments were digested by *Msc*I and *Xho*I (Takara Bio Inc, Japan), and subsequently ligated into the pET26b(+) (Novagen, USA) periplasmic expression vector using T4 DNA ligase (Takara Bio Inc, Japan). For cytoplasmic expression, the pelB signal sequence was removed by inverse PCR using the KOD enzyme (Toyobo, Japan) with the combination of a common primer, 5′-CATATGTATATCTCCTTCTTAAAGTTAAAC-3′, and either 5′-AACGATCCGTGCCCCC-3′ for *Dmel\Obp57d*, 5′-CATATGTATATCTCCTTCTTAAAGTTAAAC-3′ for *Dmel\Obp57e*, or 5′-CACAGTAATACTGCAATATTTAACC-3′ for *Dpse\Obp57de*. The resulting PCR products were self-ligated by T4 DNA ligase after phosphorylation by T4 polynucleotide kinase (Takara Bio Inc, Japan), and the insert DNAs were subcloned into the pET30b cytoplasmic expression vector (Novagen, USA) using the *Xho* I and *Xba*I sites.

### Site-directed mutagenesis of Dmel\OBP57d

Among the 16 evolutionarily conserved sites [19], 11 were selected for substitution with alanine or deletion. Site-directed mutagenesis was performed by inverse PCR using the KOD-plus enzyme and primers listed in Table 2, followed by phosphorylation of the blunt ends with T4 polynucleotide kinase and selfligation.

**Table 2:** Primer sequences for site-directed mutagenesis of Dmel\OBP57d.

Mutants	Primer sequence 5′ to 3′
**C4A**	GCCCCCCATAATCAAGGAATAGAC
	CGGATCGTTCATATGTATATCTCCT
**W22A**	GCCCCTGCAAATG
	GTCACCTAGAATTGATTCGGCTA
**P23A**	GCCGCAAATGTGGATTTGACTAGC
	CCAGTCACCTAGAATTGATTCG
**Y60A**	GCCTACGATACTGGAGTCATTGATGAA
	CTTGTCCAGAAATATCTCACCAG
**G64A**	GCCGTCATTGATGAATTGGCG
	AGTATCGTAGTACTTGTCCAGAAATATCTC
**D67A**	GCCGAATTGGCGGTGGC
	AATGACTCCAGTATCGTAGTACTTG
**A72Δ**	CCCAAAATCAATCGATGCC
	CACCGCCAATTCATCAATG
**E85A**	GCCACAGATTATTGTAGCCGAATTTT
	CATTCTAAACTCATATCGGCATC
**D87A**	GCCTATTGTAGCCGAATTTTTGC
	TGTTTCCATTCTAAACTCATATCG
**F96A**	GCCAATTGTTTAAGGCAAGAAATATTAAC
	TATAGCAAAAATTCGGCTACAATAA
**L104A**	GCCACAAAGTCATAACTCGAGCAC
	TATTTCTTGCCTTAAACAATTGAATA

### Expression and purification of recombinant proteins

BL21(DE3) *E.coli* cells (Novagen, USA) were transformed with the prepared pET30-based vector plasmids. The culture pre-incubated in LB medium with 30 µg/mL of kanamycin at 37°C overnight was inoculated into 200 mL of LB medium/kanamycin with a 1∶100 dilution, and incubated at 37°C with agitation. When the OD_600_ of the culture reached 0.6–0.8, protein expression was induced by IPTG at a final concentration of 0.4 mM, and the culture was further incubated at 37°C for 3 h. Bacterial cells were harvested by centrifugation, and mechanically disrupted by sonication at duty = 50% and power = 2 (Sonifier 250AA, Branson, USA) for 1 min×3 times with 1 min intervals in 4 mL of 20 mM Tris buffer at pH 7.4. The insoluble cytoplasmic fraction was collected by centrifugation. The pellet was washed by sonication under the same conditions, and this step was repeated twice. About 10 mg of the pellet was denatured by 10 mL of 6 M guanidine hydrochloride (or 8 M urea for Dmel\OBP57e) with 1 mM DTT at 25°C for 1 h, followed by 80-fold dilution against 20 mM Tris-HCl at pH 9.4 containing 0.1 mM of GSH and 1 mM of GSSG. Refolding was carried out by incubation at 4°C overnight. Refolded protein was loaded onto the Hitrap QHP5mL (GE healthcare, USA) after the acidity of the sample was adjusted to pH 7.4 by slowly adding 1 N HCl solution, and eluted with a linear gradient of 0–0.5 M NaCl in 20 mM Tris-HCl, pH 7.4 by using the AKTA purifier system (GE healthcare, USA). To concentrate the sample, fractions containing the target protein were pooled and loaded again onto the Hitrap QHP1mL (GE healthcare, USA), and eluted with a linear gradient of 0.1–0.4 M NaCl in the Tris buffer described above. The recombinant proteins were further purified by gel filtration with the superose-12 column (10/300 GL, GE healthcare, USA), with 0.15 M NaCl in 20 mM Tris-HCl, pH 7.4. The fractions containing the target protein were pooled and used in the subsequent assays.

### *In vitro* binding assay using intrinsic fluorescence

The concentration of the recombinant protein was determined by spectrophotometry using an extinct coefficient calculated with the ProtParam program on the ExPASy molecular biology server. E280 values were 15930, 23950, and 22460 M^−1^ cm^−1^ for Dmel\OBP57d, Dmel\OBP57e, and Dpse\OBP57de, respectively. Emission fluorescence spectra were recorded on a fluorescent spectrometer (F-2000, Hitachi, Japan). The intrinsic fluorescence was excited at 295 nm and emission spectra between 300–400 nm were recorded. A 1 µM solution of the protein in 20 mM sodium acetate, pH 5.0, was titrated with aliquots of 10 mM ligand in methanol to final concentrations of 10–100 µM, and allowed to equilibrate by incubation at 25°C for 20 min. Quenching of the intrinsic fluorescence was monitored as the decrease in relative intensity at 340 nm. The quenching value (Q) was defined as the concentration of ligand at which 3% of 1 µM OBP molecules were bound to the ligand molecules. For each OBP, the relationship between the relative intensity of fluorescence and actual amount of bound molecules was calculated using the data from the quantitative GC-MS binding assay (see below), assuming a 1∶1 association between the OBP and ligands.

### Quantitative binding assay using GC-MS

The quantification of bound ligands was performed by a modified version of a previously established method [26]. Although tridecanoic acid showed the highest affinity for these OBPs, methyl tridecanoate was used in this assay because of the simplicity of the sample treatment. Conditions were the same as those for the intrinsic fluorescence assay, i.e. 90 µM methyl tridecanoate in methanol was incubated with 1 µM OBP in 1 mL of 20 mM sodium acetate, pH 5.0 for 30 min. Unbound ligand was washed out by ultrafilteration (Amicon Ultra 0.5 mL 10 K, Millipore, USA), and the retentate (80 µL) was transferred to a 0.5-mL tube. Again 20 µL of the buffer was added onto the filter to wash it, and then pooled in the same tube. To release the bound ligand, 50 µL of 1 M Tris-HCl, pH 9.4, was added, and incubated for 5 min. Before extraction of the released ligand by 100 µL of n-hexane, 500 pmol of methyl dodecanoate was added as an internal standard. The organic solvent layer was transferred into a new micro tube, and the solvent was evaporated completely using a spin drier. The dried sample was dissolved in 10 µL of ethanol, and analyzed by gas chromatography mass spectrometer (GCMS-QP5050A, Shimadzu, Japan) equipped with a capillary column (HP-INNOWAX, 30 m, 0.25 mm, 0.25 µm, Agilent Technologies, USA) using the following temperature program: 60°C for 4 min, increased to 200°C at a rate of 10°C/min, and held at the final temperature for 10 min. The amount of ligand was calculated by counting the area of the target peak.

### Oviposition site selection assay

*D.melanogaster w^1118^* was used as the wild-type. The OBP knockout strains (*Obp57d^KO^, Obp57e^KO^, and Obp57d+e^KO^*) were described previously [16]. Newly eclosed adults were collected in a vial containing standard cornmeal-based fly food and grains of dry yeast, and staged for 3 days at 25°C under a 12 h∶12 h light/dark cycle. Before each assay, the staged flies were incubated overnight in a vial containing wet filter paper and yeast paste. The oviposition medium was composed of 1% ethanol, 1% sucrose, 0.05% methyl cellulose, 0.05% dry yeast, and 0.8% agar. Tridecanoic acid-containing medium (1 mM) was prepared as follows: 11.9 µl of tridecanoic acid was dissolved in 500 µL of ethanol, then added to 5 ml of a 0.5% methyl cellulose (400 cP) solution (Wako pure chemical, Japan) and mixed well by pipetting. The resulting tridecanoic acid suspension was dispensed (25 µL per well) into a flexible 96-well assay plate (Falcon #353911, Becton Dickenson Co., USA). To each well, 100 µL of a 2.5% sucrose solution was added, and then mixed gently by vortexing. Then, 125 µL of hot agar-yeast solution (1.6% Bacto agar, Becton Dickenson Co., USA; 0.1% dry yeast, Oriental Yeast Co., Japan) was added and again gently mixed by vortexing. After the medium had solidified, the assay plate was cut into 2×2 pieces, each of which was placed in a glass vial with a disc of wet filter paper. A staged, single female was introduced to the vial and allowed to lay eggs overnight. Each assay started approximately at Zeitgeber time (ZT) 8 and ended at ZT-2, with the light phase from ZT-0 to ZT-12. The number of eggs laid on the medium was counted, and a preference index (PI) was calculated as; PI = (*N_acid_*−*N_cont_*)/(*N_acid_*+*N_cont_*) where *N_acid_* and *N_cont_* represent the number of eggs on the tridecanoic acid-containing and control medium, respectively. PI was calculated for each individual. A single set of assays was carried out with 8 females, and repeated 6 times on independent days using independently prepared flies. A total of 48 individuals were analyzed for each strain. Means of PI were analyzed for differences between genotypes using the Kruskal-Wallis test, followed by the pairwise Wilcoxon rank sum test with p-value adjustment by Holm's method for multiple comparisons.

## Supporting Information

Figure S1 **Purification of monomeric OBPs by anion exchange chromatography.** (A–C) Fractions eluted by anion exchange chromatography were loaded on a SDS-PAGE gel without reducing agents. Red and blue boxes indicate monomeric and multimeric OBPs, respectively. (A) Dmel\OBP57d, (B) Dmel\OBP57e and (C) Dpse\OBP57de. (D) Purified OBPs were examined by native-PAGE.
(PDF)

Figure S2 **Confirmation of the purity of Dmel\OBP57d, Dmel\OBP57e and Dpse\OBP57de by HPLC.** The purified proteins were analyzed by HPLC (see Structural analyses in this document). Only a single peak was observed for Dmel\OBP57d (A), Dmel\OBP57e (B) and Dpse\OBP57de (C), suggesting that a single form of protein was recovered.
(PDF)

Figure S3 **Effect of pH on the biochemical characteristics of Dmel\OBP57d.** (A) Secondary structure of Dmel\OBP57d was examined using Far-UV circular dichroism spectra at pH 5.0 (red) and pH 7.4 (blue). Dmel\OBP57d is comprised of α helices, whose secondary structure was not affected by the acidity of the buffer. (B) Dmel\OBP57d showed higher affinity to tridecanoic acid at pH 5.0 (red) than at pH 7.4 (blue).
(PDF)

Figure S4 **Binding affinity of Dmel\OBP57d to the ligands of other OBPs.** Binding affinity of Dmel\OBP57d to hexyl benzoate (HB), 2-pentadecanone (2PO) and linalool (LL) was examined. (A) Relative fluorescence intensity. (B) Comparisons of the binding affinity using the Q value. All of these compounds showed lower affinity than tridecanoic acid.
(PDF)

Table S1 **Summary of mass analysis.** Correspondence of the observed fragments to the theoretical ones. Although not all of the theoretical fragments were observed, fragments suggesting non-conventional S-S bonds were not observed for all of the three OBPs.
(PDF)

Materials and Methods S1 **Structural analyses.** Methods for the analyses shown in Fig. S1, S2, S3, and S4 and Table S1.
(PDF)
