# Supplementary figures and images for: Functional Evolution of Duplicated Odorant-Binding Protein Genes, Obp57d and Obp57e, in Drosophila
Source: PLoS One. 2012 Jan 6;7(1):e29710. doi: 10.1371/journal.pone.0029710 (PMC3253112; doi:10.1371/journal.pone.0029710)

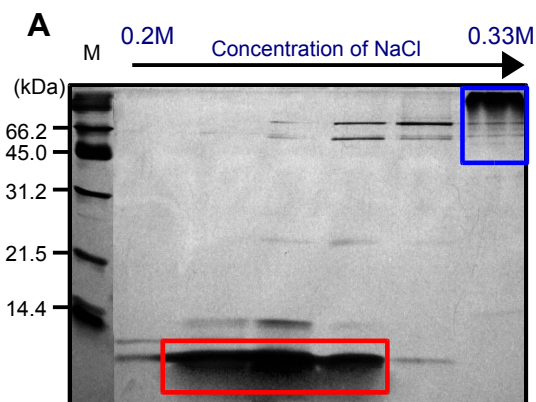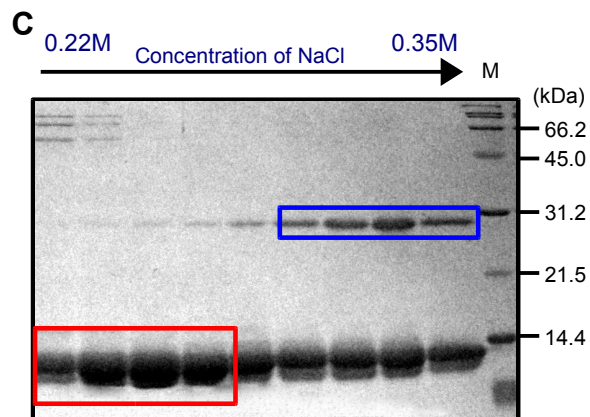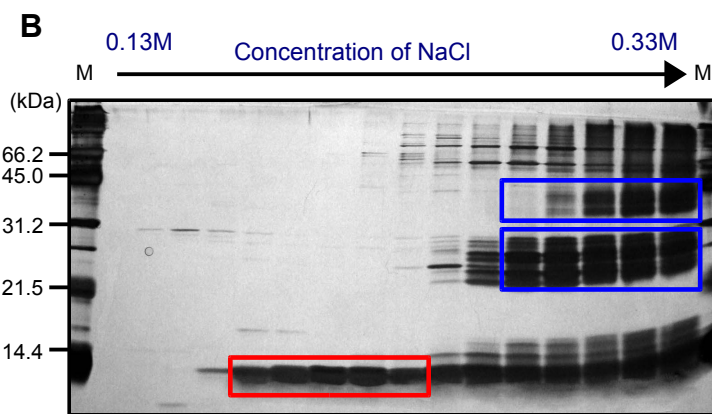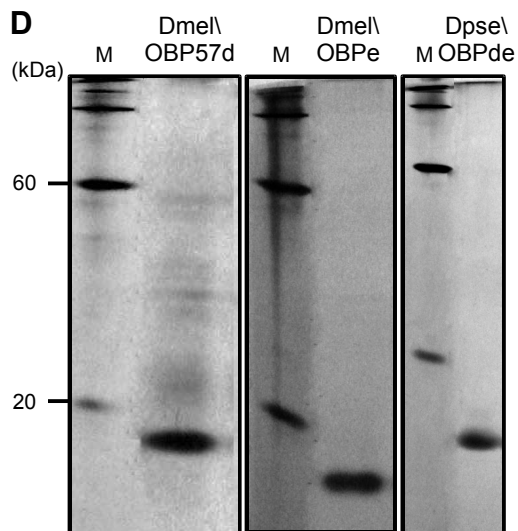

Supplement: Figure S1 — Purification of monomeric OBPs by anion exchange chromatography. (A–C) Fractions eluted by anion exchange chromatography were loaded on a SDS-PAGE gel without reducing agents. Red and blue boxes indicate monomeric and multimeric OBPs, respectively. (A) Dmel\OBP57d, (B) Dmel\OBP57e and (C) Dpse\OBP57de. (D) Purified OBPs were examined by native-PAGE. (PDF) [file pone.0029710.s001.pdf]

**A**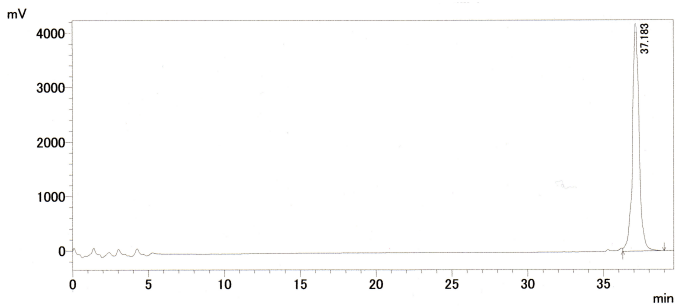**B**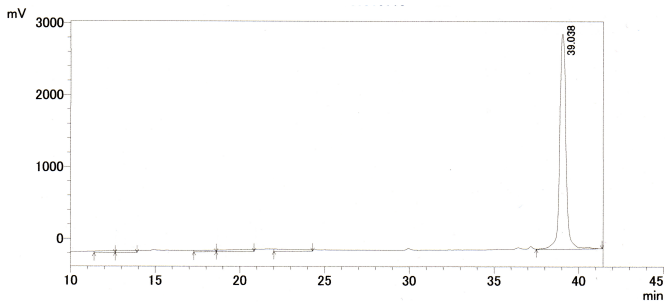**C**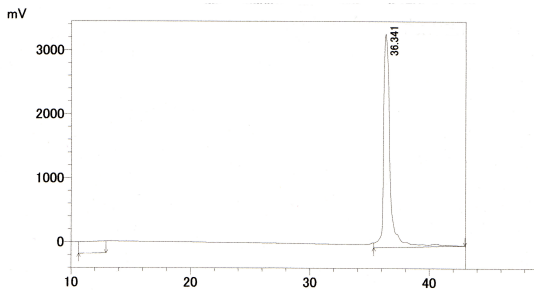

Supplement: Figure S2 — Confirmation of the purity of Dmel\OBP57d, Dmel\OBP57e and Dpse\OBP57de by HPLC. The purified proteins were analyzed by HPLC (see Structural analyses in this document). Only a single peak was observed for Dmel\OBP57d (A), Dmel\OBP57e (B) and Dpse\OBP57de (C), suggesting that a single form of protein was recovered. (PDF) [file pone.0029710.s002.pdf]

**A**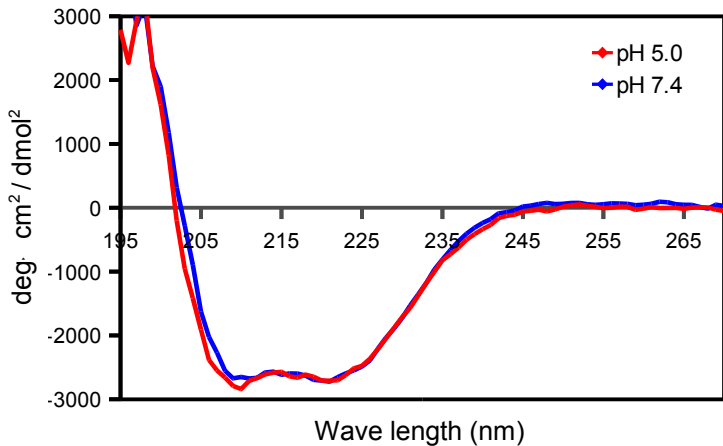**B**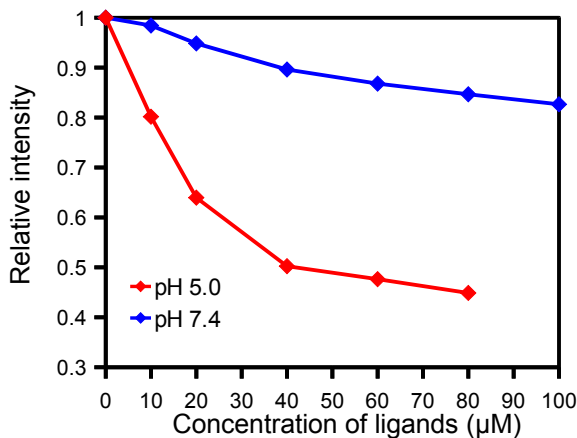

Supplement: Figure S3 — Effect of pH on the biochemical characteristics of Dmel\OBP57d. (A) Secondary structure of Dmel\OBP57d was examined using Far-UV circular dichroism spectra at pH 5.0 (red) and pH 7.4 (blue). Dmel\OBP57d is comprised of α helices, whose secondary structure was not affected by the acidity of the buffer. (B) Dmel\OBP57d showed higher affinity to tridecanoic acid at pH 5.0 (red) than at pH 7.4 (blue). (PDF) [file pone.0029710.s003.pdf]

**A**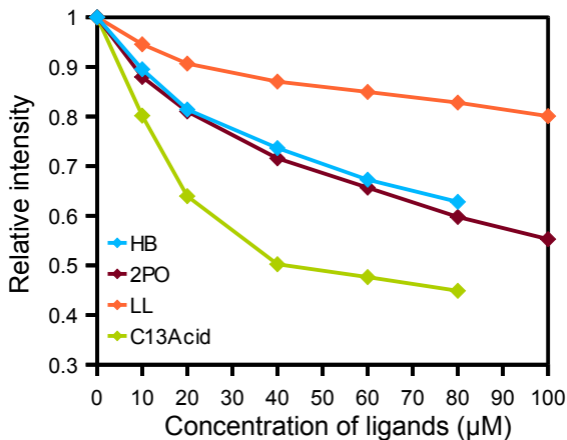**B**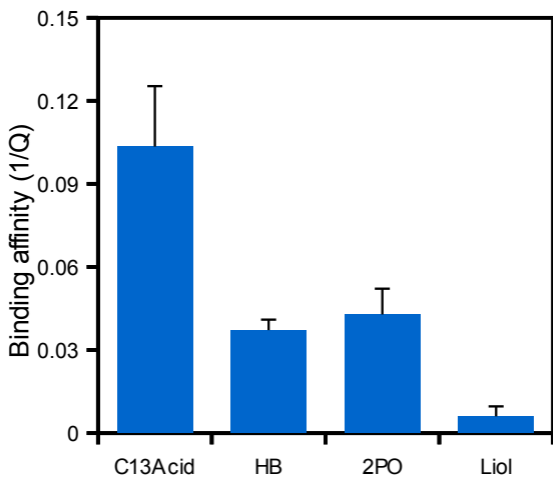

Supplement: Figure S4 — Binding affinity of Dmel\OBP57d to the ligands of other OBPs. Binding affinity of Dmel\OBP57d to hexyl benzoate (HB), 2-pentadecanone (2PO) and linalool (LL) was examined. (A) Relative fluorescence intensity. (B) Comparisons of the binding affinity using the Q value. All of these compounds showed lower affinity than tridecanoic acid. (PDF) [file pone.0029710.s004.pdf]
