# Supplementary material for: Functional Evolution of Duplicated Odorant-Binding Protein Genes, Obp57d and Obp57e, in Drosophila
Source: PLoS One. 2012 Jan 6;7(1):e29710. doi: 10.1371/journal.pone.0029710 (PMC3253112; doi:10.1371/journal.pone.0029710)
Supplement: Table S1 — Summary of mass analysis. Correspondence of the observed fragments to the theoretical ones. Although not all of the theoretical fragments were observed, fragments suggesting non-conventional S-S bonds were not observed for all of the three OBPs. (PDF) [file pone.0029710.s005.pdf]

protein	endopeptidase	Observed m/z	charge	mass	theoretical mass	residue number	S-S Pair	instrument
	trypsin	7281.02	1+	7281.02	7281.16	1-33, 34-56, 59-66	Cys1- 2- 3- 5	MALDI
Dmel\OBP57d	trypsin	686.30	2+	1371.60	1371.70	57-58, 67-75	Cys4 - 6	LC-MS
	Chymotrypsin	698.19	1+	698.19	698.32	1-6		LC-MS
	Chymotrypsin	862.36	2+	1723.72	1723.73	37-39, 84-94	Cys2 – 5	LC-MS
	V8 endopeptidase	2722.74	1+	2722.74	2721.38	53-75		MALDI
Dmel\OBP57e	V8 endopeptidase	916.70	3+	2748.10	2748.41	76-82, 92-108	Cys4 – 6	LC-MS
	Chymotrypsin	816.33	2+	1631.66	1631.74	9-16, 46-51	Cys1 – 3	LC-MS
	V8 endopeptidase	823.86	2+	1646.72	1646.87	22-34		LC-MS
	Chymotrypsin	743.72	2+	1486.44	1486.52	43-45, 89-97	Cys2 – 5	LC-MS
Dpse\OBP57d	V8 endopeptidase	3674.23	1+	3674.23	3675.19	75-87, 97-115	Cys4 – 6	MALDI
