# Supplementary material for: Functional Evolution of Duplicated Odorant-Binding Protein Genes, Obp57d and Obp57e, in Drosophila
Source: PLoS One. 2012 Jan 6;7(1):e29710. doi: 10.1371/journal.pone.0029710 (PMC3253112; doi:10.1371/journal.pone.0029710)
Supplement: Materials and Methods S1 — Structural analyses. Methods for the analyses shown in Fig. S1, S2, S3, and S4 and Table S1. (PDF) [file pone.0029710.s006.pdf]

Materials and Methods S1

Structural analyses

The recombinant proteins were further purified by HPLC (Prominence UPLC, Shimadzu) with a C8 column (UG 120, 4.6 mm x 150 mm, Shiseido, Japan) and linear gradient of 0 – 80 % of acetonitrile containing TFA at 0.01 %. Eluted samples were lymphonized and dissolved into the digestion buffer (see below). Ten micrograms of protein in the digestion buffer was incubated at 100 °C for 5 min, and immediately incubated on ice for 2 min. Then, endopeptidase was added and incubated at 37 °C overnight. Dmel\OBP57d was digested by trypsin (1:100 ratio) and chymotrypsin (1:20 ratio), in 50 mM Tris-HCl, pH 8.0, and in 50 mM ammonium bicarbonate, pH 7.8, respectively. For the digestion of Dmel\OBP57e and Dpse\OBP57de, V8 endopeptidase was used instead of trypsin. Molecular mass of the resulting fragments was determined by liquid chromatography mass spectrometry (LC-MS) and matrix-assisted laser desorption (MALDI). LC was equipped with a direct nano-flow pump with a pressure limit of ~300 bars (LC Assist, Tokyo, Japan) which delivers solvent to the fritless spray tip ESI column, a ReNCon gradient device [1]. Mass spectrometer was operated by Q-Tof Ultima (Waters, Bedford, USA). Molecular mass of larger fragments was determined by MALDI TOF/TOF 4800 (Applied Biosystems, USA). CD spectra were recorded with a J-715 spectropolarimeter (JASCO Corp., USA). Dmel\OBP57d (0.3 mg/ml) was prepared either in 20 mM ammonium acetate, pH 5.0 or in 20 mM Tris-HCl, pH 7.4.

Reference

1. Taoka M, Yamauchi Y, Nobe Y, Masaki S, Nakayama H, Ishikawa H, Takahashi N, Isobe T. (2009) An analytical platform for mass spectrometry-based identification and chemical analysis of RNA in ribonucleoprotein complexes. Nucl Acid Res 37: e140.
